# Utilization of Point-of-Care Ultrasound as an Imaging Modality in the Emergency Department: A Systematic Review and Meta-Analysis

**DOI:** 10.7759/cureus.52371

**Published:** 2024-01-16

**Authors:** Apurva Popat, Samyuktha Harikrishnan, Niran Seby, Udvas Sen, Sagar K Patel, Lakshay Mittal, Mitkumar Patel, Charitha Vundi, Yashasvi Patel, Babita, Ashish Kumar, Akash A Nakrani, Mahir Patel, Sweta Yadav

**Affiliations:** 1 Internal Medicine, Marshfield Clinic Health System, Marshfield, USA; 2 Internal Medicine, Gulf Medical University, Ajman, ARE; 3 Internal Medicine, Tbilisi State Medical University, Tbilisi, GEO; 4 Internal Medicine, Agartala Government Medical College, Agartala, IND; 5 Internal Medicine, Gujarat Adani Institute of Medical Sciences, Bhuj, IND; 6 Internal Medicine, Pandit Bhagwat Dayal Sharma Post Graduate Institute of Medical Sciences, Rohtak, IND; 7 Internal Medicine, Mahatma Gandhi Mission (MGM) Medical College, Navi Mumbai, IND; 8 Internal Medicine, Konaseema Institute of Medical Sciences and Research Foundation, Amalapuram, IND; 9 Internal Medicine, Geetanjali Medical College and Hospital, Udaipur, IND; 10 Internal Medicine, Uzhhorod National University, Uzhhorod, UKR; 11 General Practice, Gujarat Medical Education and Research Society (GMERS) Medical College and Hospital, Ahmedabad, IND; 12 Internal Medicine, Gujarat Adani Institute of Medical Sciences, Surat, IND; 13 Medical School, Byramjee Jeejeebhoy Medical College, Ahmedabad, IND; 14 Internal Medicine, Gujarat Medical Education and Research Society (GMERS) Medical College and Hospital, Ahmedabad, IND

**Keywords:** pocus efficiency studies, emergency medical technology, ultrasound in clinical care, pocus diagnostic accuracy, clinical procedure assistance, emergency department diagnostics, radiological imaging techniques, procedure guidance ultrasound, diagnostic ultrasound applications, pocus in emergency medicine

## Abstract

Point-of-care ultrasound (POCUS) is an imaging modality that has become a fundamental part of clinical care provided in the emergency department (ED). The applications of this tool in the ED have ranged from resuscitation, diagnosis, and therapeutic to procedure guidance. This review aims to summarize the evidence on the use of POCUS for diagnosis and procedure guidance. To achieve this, CrossRef, PubMed, Cochrane Library, Web of Science, and Google Scholar databases were extensively searched for studies published between January 2000 and November 2023. Additionally, the risk of bias assessment was performed using the Quality Assessment of Diagnostic Accuracy Studies 2 (for studies on the diagnostic role of POCUS) and Cochrane Risk of Bias tool (for studies on the use of POCUS for procedure guidance). Furthermore, diagnostic accuracy outcomes were pooled using STATA 16 software (StatCorp., College Station, TX, USA), while outcomes related to procedure guidance were pooled using the Review Manager software. The study included 81 articles (74 evaluating the diagnostic application of POCUS and seven evaluating the use of POCUS in guiding clinical procedures). In our findings sensitivities and specificities for various conditions were as follows: appendicitis, 65% and 89%; hydronephrosis, 82% and 74%; small bowel obstruction, 93% and 82%; cholecystitis, 75% and 96%; retinal detachment, 94% and 91%; abscess, 95% and 85%; foreign bodies, 67% and 97%; clavicle fractures, 93% and 94%; distal forearm fractures, 97% and 94%; metacarpal fractures, 94% and 92%; skull fractures, 91% and 97%; and pleural effusion, 91% and 97%. A subgroup analysis of data from 11 studies also showed that the two-point POCUS has a sensitivity and specificity of 89% and 96%, while the three-point POCUS is 87% sensitive and 92% specific in the diagnosis of deep vein thrombosis. In addition, the analyses showed that ultrasound guidance significantly increases the overall success rate of peripheral venous access (p = 0.02) and significantly reduces the number of skin punctures (p = 0.01) compared to conventional methods. In conclusion, POCUS can be used in the ED to diagnose a wide range of clinical conditions accurately. Furthermore, it can be used to guide peripheral venous access and central venous catheter insertion.

## Introduction and background

Ultrasound is a technology employed in various medical fields, including obstetrics, cardiology, critical care, emergency medicine, pediatrics, and primary care. In recent years, ultrasound technology has evolved from large immobile machines to portable devices on wheeled carts and currently to handheld devices capable of fitting in the clinician’s pocket [1]. Point-of-care ultrasound (POCUS) is primarily used to complement physical examination. It is gaining attention because it can enable disease screening, accelerate definitive diagnosis, guide clinical decision-making, and decrease overall healthcare expenses [2-5]. Currently, there are many POCUS devices in the healthcare field, with research suggesting that these devices are highly accurate when employed by trained personnel and often rival or surpass other imaging modalities [6-8].

According to the American College of Emergency Physicians, the use of POCUS in emergency medicine is categorized into five clinical categories, i.e., resuscitative, diagnostic, symptom or sign-based, procedure guidance, and therapeutic [9]. In resuscitation, POCUS has been reported to serve as a prognostic tool in cardiac arrest, where physical examination is not always accurate [10,11]. In addition, POCUS can aid in the rapid determination of the underlying causes of undifferentiated shock, allowing for targeted and effective resuscitation efforts. As a diagnostic tool, POCUS is versatile as it can help healthcare providers evaluate various organ systems at the patients’ bedside. Furthermore, it has proven effective in enhancing safe and accurate invasive procedures within the emergency department (ED). These procedures vary from central venous placement and draining abscess to assisting regional nerve blocks [12,13].

Although POCUS has a wide range of applications in the ED, we decided to focus on the following applications: the use of POCUS in diagnosing various clinical conditions and guiding clinical procedures.

## Review

Methodology

Information Sources and Searches

The CrossRef, PubMed, Cochrane Library, Web of Science, and Google Scholar databases were extensively searched for primary studies published between January 2000 and November 2023. In addition, the bibliographies of potential studies were reviewed for additional studies. The strategy employed to identify studies from the databases mentioned above was as follows: (Point-of-care ultrasound OR POCUS OR handheld ultrasound OR pocket-size ultrasound OR bedside ultrasound OR Emergency ultrasound) AND (Emergency department OR ED OR Emergency medicine OR emergency room OR emergency ward). Furthermore, all gray literature was avoided as it includes unpublished data likely to undermine the scientific purpose and statistical power of this study.

Eligibility Criteria

Two impartial reviewers analyzed the studies from the aforementioned databases, included articles if they were published in English, and evaluated the use of POCUS in the ED. On the other hand, articles that did not meet these criteria or were designed as conference abstracts, reviews, case reports, and letters to the editors were excluded. Furthermore, studies reporting the use of POCUS by prehospital emergency medical services were excluded. Any discrepancy during this process was amicably resolved via constructive debates between the two reviewers.

Data Extraction

Two independent reviewers analyzed the eligible studies and abstracted the data required for review and analysis into separate Excel files. In case of disparities in the abstracted data, the reviewers engaged in constructive discussions, and if they could not reach a compromise, a third reviewer was consulted. The data retrieved by these reviewers included author ID (surname of the primary author and year of publication), study design, pertinent characteristics of enrolled patients (sample size and sex distribution), the use of POCUS, test condition, reference/criterion standard, operators, and the outcomes.

The outcomes in our study were divided into procedural and diagnostic. The diagnostic outcomes included sensitivity and specificity, while the procedural outcomes were success rates, procedure-related complications, time to successful catheter insertion, and the average number of skin punctures.

Quality Appraisal

The methodological quality of studies in the present review was assessed using two different tools. The Quality Assessment of Diagnostic Accuracy 2 (QUADAS-2) tool embedded within the Review Manager software was used to evaluate the risk of bias in each study assessing the diagnostic role of POCUS. Using this tool, the included studies were assessed according to four bias assessment domains (patient selection, index test, reference standard, and flow and timing) and three applicability domains (patient selection, index test, and reference standard).

On the other hand, bias assessment of studies on the use of POCUS to guide procedures in the ED was done using the Cochrane Risk of Bias tool (RoB). With this tool, the risk of bias in each study was assessed according to the selection, attrition, performance, reporting, and other biases. A low risk of bias meant the criteria in each domain were sufficiently addressed, while a high risk of bias meant that the criteria were not addressed. On the other hand, an unclear risk of bias referred to the inability of reviewers to provide conclusive judgment due to insufficient information.

Data Synthesis

STATA software (StataCorp Stata MP 16.0, College Station, TX, USA) was used to pool the outcomes on the diagnostic accuracy of POCUS. On the other hand, the Review Manager software (RevMan 5.4.1) was used to pool outcomes related to the use of POCUS in guiding procedures. The DerSimonian Laird effects model was employed in all statistical analyses to calculate conservative effect sizes and counter the anticipated heterogeneity. Furthermore, the heterogeneity was calculated using the I^2^ statistics, wherein values above 50% were regarded as substantial [14]. For dichotomous outcomes, the effect size was calculated using the simple odds ratio (OR), and the mean difference (MD) calculations were used to pool the continuous outcomes. In cases where median, range, and interquartile ranges were presented, the formula described by Hozo and colleagues was used to calculate the means and standard deviations [15].

Results

Study Selection

**Figure 1:**
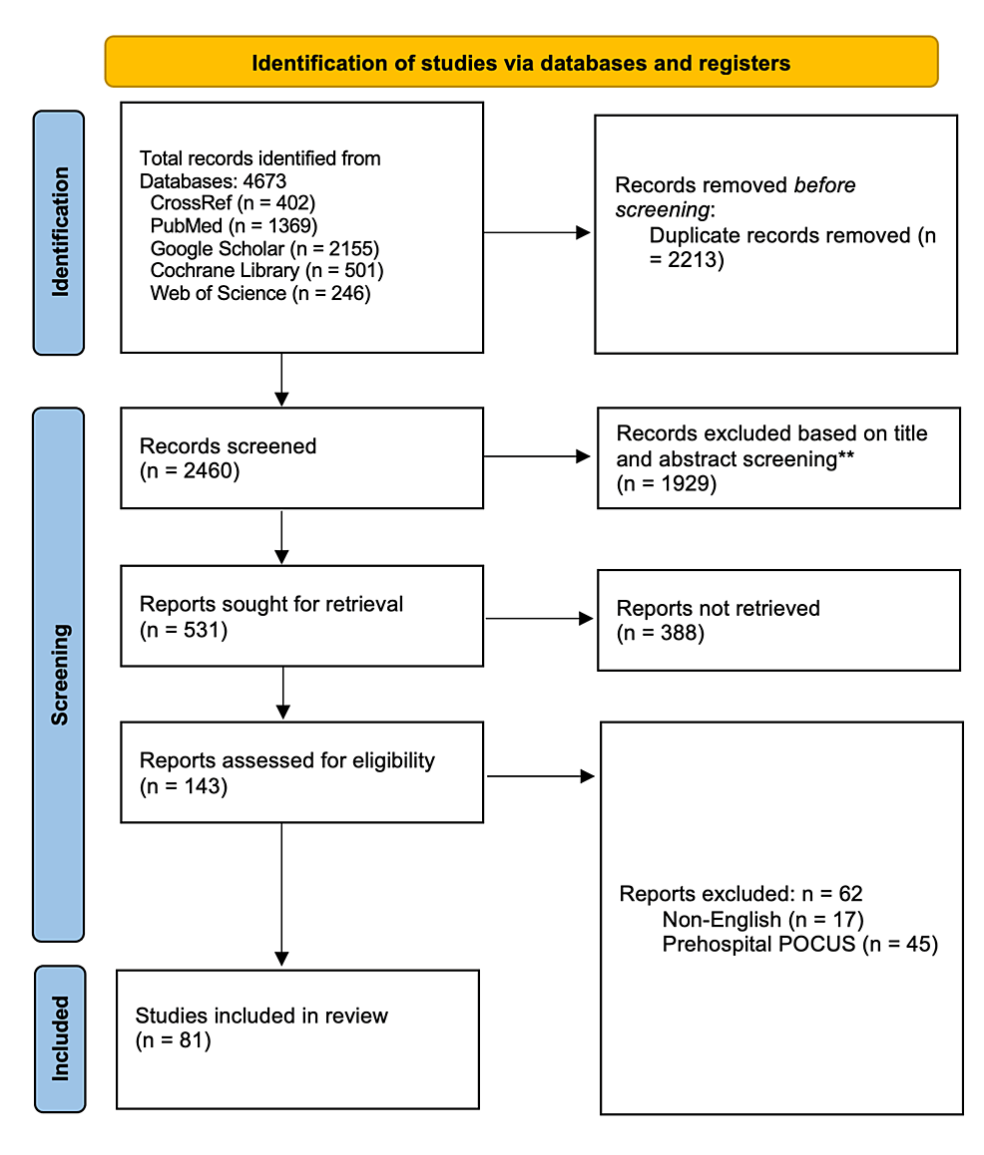
Preferred Reporting Items for Systematic Reviews and Meta-Analyses flow diagram for study selection.

Our preliminary database search identified 4,673 viable records. The in-depth duplicate screening criteria led to the exclusion of 2,213 records regarded as exact or nearly identical duplicates. Additionally, the title and abstract screening led to the exclusion of 1,929 records. Of the remaining 531 articles, 388 were not retrieved as they were either non-full-text records, ongoing trials, conference abstracts, case reports, experimental studies, letters to the editor, or systematic reviews and meta-analyses. Finally, only 81 articles were eligible for inclusion, while the other 62 were excluded as follows: 17 were published in different languages, and 45 evaluated the prehospital use of POCUS. Figure 1 illustrates the Preferred Reporting Items for Systematic Reviews and Meta-Analyses flow diagram of study selection for the systematic review.

Summary of Study Characteristics

Of the 81 included studies, 74 evaluated the use of POCUS as a diagnostic tool and seven assessed the use of POCUS for procedure guidance. Furthermore, 29 reported the diagnostic utility of abdominal POCUS, five ocular POCUS, six soft tissue POCUS, 19 musculoskeletal POCUS, 11 vascular POCUS, and four lung POCUS (Table 1).

**Table 1:** Summary of study characteristics on the use of POCUS as a diagnostic tool in the emergency department. NR = not reported; CT = computed tomography; RUS = radiologist-performed ultrasound; FB = foreign body; SBO = small bowel obstruction; I&D = incision and drainage; DUS = duplex ultrasound; VDUS = venous duplex ultrasound; POCUS = point-of-care ultrasound

Author ID	Study design	Participant characteristics		Condition	Reference/criterion standard	Outcomes	
		Sample (n)	M/F			Sensitivity (%)	Specificity (%)
Elikashvili et al. (2014) [16]	Prospective observational study	150	66/84	Appendicitis	Surgical or pathological findings	60	94
Gungor et al. (2017) [17]	Prospective observational study	264	151/113		Surgical or pathological findings or CT scans	92.3	95.8
Nicole et al. (2018) [18]	Prospective observational study	117	65/52		Surgical or pathological findings	53	82
Becker et al. (2022) [19]	Prospective observational study	256	127/129		Pathological findings	85	63
Fox et al. (2007) [20]	Retrospective cohort study	155	85/70		RUS, CT scan, or pathological findings	39	90
Atalar et al. (2022) [21]	Prospective observational study	123	69/54		Pathological findings	73.3	89.2
Fathi et al. (2014) [22]	Prospective observational study	97	56/41		Pathological findings	44.18	85.18
Mallin et al. (2015) [23]	Prospective observational study	97	NR		Surgical or pathological findings	67.7	98.4
Sharif et al. (2018) [24]	Prospective observational study	90	NR		Pathology, laparoscopy, CT scan, or RUS	69.2	90.6
Fox et al. (2008) [25]	Prospective cohort study	132	67/65		Pathological findings	65	90
Abgottspon et al. (2022) [26]	Retrospective study	61	-/61		Pathological findings	66.7	96.8
Gaspari & Horst (2005) [27]	Prospective observational study	102	45/57	Hydronephrosis	CT scan	86.8	82.4
Pathan et al. (2018) [28]	Prospective observational study	651	545/106		CT scan	85.7	65.9
Guedj et al. (2015) [29]	Prospective observational study	433	171/262		NR	76.5	97.2
Herbst et al. (2014) [30]	Prospective observational study	670	325/345		CT scan	72.6	73.3
Javaudin et al. (2017) [31]	Prospective observational study	50	25/25		RUS	100	71
Sibley et al. (2020) [32]	Prospective observational study	413	208/205		CT scan	77.1	71.8
Al-Balushi et al. (2022) [33]	Prospective cross-sectional study	303	247/56		CT scan	75.8	55.2
Riddell et al. (2014) [34]	Retrospective study	125	79/46		CT scan	78.4	-
Boniface et al. (2020) [35]	Prospective observational study	125	58/67	SBO	CT scan	87.5	75.3
Unleur et al. (2010) [36]	Prospective observational study	174	106/68		Surgical pathology or CT scan	97.7	92.7
Becker et al. (2019) [37]	Prospective observational study	217	103/114		CT scan	88	54
Biggs et al. (2022) [38]	Prospective cohort study	101	35/66		CT scan	90	91.7
Jang et al. (2011) [39]	Prospective observational study	76	NR		CT scan	93.9	81.4
Frasure et al. (2018) [40]	Retrospective cohort study	64	22/42		CT scan or final diagnosis	94.3	95.2
Shekarchi et al. (2018) [41]	Observational diagnostic study	342	126/216	Cholecystitis	RUS	89.58	96.59
Sharif et al. (2021) [42]	Retrospective study	577	NR		Pathology, laparoscopy, or CT scan	67.1	97.6
Summers et al. (2010) [43]	Prospective observational study	164	45/119		Surgical pathology	87	82
Wehrle et al. (2022) [44]	Retrospective study	147	41/106		Final diagnosis	40	99
Yoonesi et al. (2010) [45]	Prospective observational study	48	26/22	Retinal detachment	Final diagnosis	100	83
Kim et al. (2019) [46]	Prospective study	115	41/74		Final diagnosis	75	94
Chu et al. (2017) [47]	Prospective observational study	139	47/92		Final diagnosis	88	87
Lahham et al. (2019) [48]	Prospective observational study	225	135/90		Final diagnosis	96.9	88.1
Jacobsen et al. (2016) [49]	Prospective study	109	52/57		Final diagnosis	91	96
Mower et al. (2019) [50]	Prospective observational study	1216	708/508	Abscess	Positive I&D	94	94.1
Squire et al. (2005) [51]	Prospective clinical trial	107	74/33		Positive I&D	98	88
Adams et al. (2016) [52]	Prospective study	151	67/84		Needle aspiration or positive I&D	96	87
Iverson et al. (2012) [53]	Prospective study	65	23/42		Positive I&D	97.5	69.2
Sivitz et al. (2010) [54]	Prospective observational study	50	29/21		Needle aspiration or positive I&D	90	83
Friedman et al. (2005) [55]	Prospective cohort study	105	57/48	FB	Radiography	66.7	96.6
Cross et al. (2010) [56]	Prospective study	100	75/25	Clavicle fractures	Radiography	95	96
Chien et al. (2011) [57]	Prospective study	58	39/19		Radiography	89.7	89.5
Waterbrook et al. (2013) [58]	Prospective observational study	103	52/51		Radiography	100	100
Sivrikaya et al. (2016) [59]	Cross-sectional study	90	43/47	Distal forearm fracture	Radiography or CT scan	97.4	92.6
Poonai et al. (2017) [60]	Cross-sectional study	169	88/81		Radiography	94.7	93.5
Laka et al. (2017) [61]	Prospective observational study	115	58/57		Radiography	94.4	96.8
Chaar-Alvarez et al. (2011) [62]	Prospective study	108	NR		Radiography	96	93
Epema et al. (2019) [63]	Prospective study	100	50/50		Radiography	95	86
Kozaci et al. (2015) [64]	Prospective observational study	83	65/18		Radiography	98	96
Wood et al. (2021) [65]	Prospective observational study	47	13/34		Radiography	100	100
Kocaoglu et al. (2016) [66]	Prospective single-blinded RCT	96	NR	Metacarpal fractures	Radiography	92.5	98.28
Aksay et al. (2015) [67]	Prospective study	81	70/11		Radiography	97.4	92.9
Kozaci et al. (2015) [68]	Prospective observational study	66	59/7		Radiography	92	87
Masaeli et al. (2019) [69]	Prospective cross-sectional study	538	295/243	Skull fractures	CT scan	92.3	95.8
Choi et al. (2020) [70]	Prospective observational study	87	42/45		CT scan	76.9	100
Parri et al. (2018) [71]	Prospective observational study	115	62/53		CT scan	90.9	85.2
Rabiner et al. (2013) [72]	Prospective observational study	69	45/24		CT scan	88	97
Riera & Chen (2012) [73]	Prospective study	46	NR		CT scan	82	94
Weinberg et al. (2010) [74]	Prospective cohort study	212	NR	Skull, clavicle, and metacarpal fractures	Radiography or CT scan	100, 89, and 80	100, 83, and 85
Canakci et al. (2020) [75]	Retrospective study	266	124/142	DVT	RUS and venography	93	93
Garcia et al. (2018) [76]	Prospective cross-sectional study	109	49/60		RUS	93.2	90
Jang et al. (2004) [77]	Prospective study	72	24/48		Contrast venography, CT venography or VDUS	100	91.8
Crisp et al. (2010) [78]	Prospective cross-sectional study	188	NR		RUS	100	99.4
Jahanian et al. (2019) [79]	Prospective cross-sectional study	72	36/36		RUS	53.8	85.7
Frazee et al. (2001) [80]	Prospective observational study	76	48/28		DUS	88.9	75.9
Dehbozorgi et al. (2019) [81]	Prospective cross-sectional study	240	120/120		DUS	100	93.33
Kline et al. (2008) [82]	Prospective study	185	76/109		RUS	70	89
Pujol et al. (2018) [83]	Prospective study	56	23/33		VDUS	100	100
Zitek et al. (2016) [84]	Prospective study	234	119/115		RUS	57.1	96.1
Zuker-Herman et al. (2018) [85]	Prospective study	285	77/118		DUS	90.57	98.52
Baid et al. (2022) [86]	Prospective observational study	237	142/95	Pleural effusion	Final diagnosis	100	97.7
Zanobetti et al. (2011) [87]	Prospective observational study	404	206/198		CT scan	90	73
Zanobetti et al. (2017) [88]	Prospective observational study	2,683	1367/1316		Final diagnosis	77.6	99.2
Buhumaid et al. (2019) [89]	Prospective observational study	128	71/57		Final diagnosis	100	71

On the other hand, five of the seven studies reporting the use of POCUS for procedure guidance were about peripheral venous access and the other two were about central venous access (Table 2).

**Table 2:** Summary of study characteristics on the use of POCUS to guide procedures in the ED. NR = not reported; ED = emergency department; EP = emergency physician; RCT = randomized controlled trial; POCUS = point-of-care ultrasound

Author ID	Study design	Patient characteristics		Procedure	Operator	Outcomes
		Sample	M/F			
Costantino et al. (2005) [90]	Prospective, non-blinded systematically allocated study	61	NR	Peripheral intravenous access	EPs and residents	Success rate, attempts, time to successful cannulation, and complications
Costantino et al. (2010) [91]	Prospective non-blinded RCT	60	23/37		Emergency residents	Success rate, attempts, time to successful cannulation, and complications
Doniger et al. (2009) [92]	Prospective RCT	50	25/25		Emergency medicine nurse	Success rate, attempts, time to successful cannulation, and complications
Stein et al. (2009) [93]	Prospective non-blinded RCT	59	21/38		EP	Attempts and time to successful cannulation
Bauman et al. (2009) [94]	Prospective, non-blinded systematically allocated study	75	21/54		ED technicians	Success rate, time to successful cannulation, attempts, and complications
Miller et al. (2002) [95]	Prospective study	122	NR	Central venous access	EP	Time to successful cannulation, attempts, and complications
Gallagher et al. (2014) [96]	Retrospective cohort study	168	80/88		EPs, fellows, or residents	Success rate and complications

Risk of Bias Assessment

**Figure 2:**
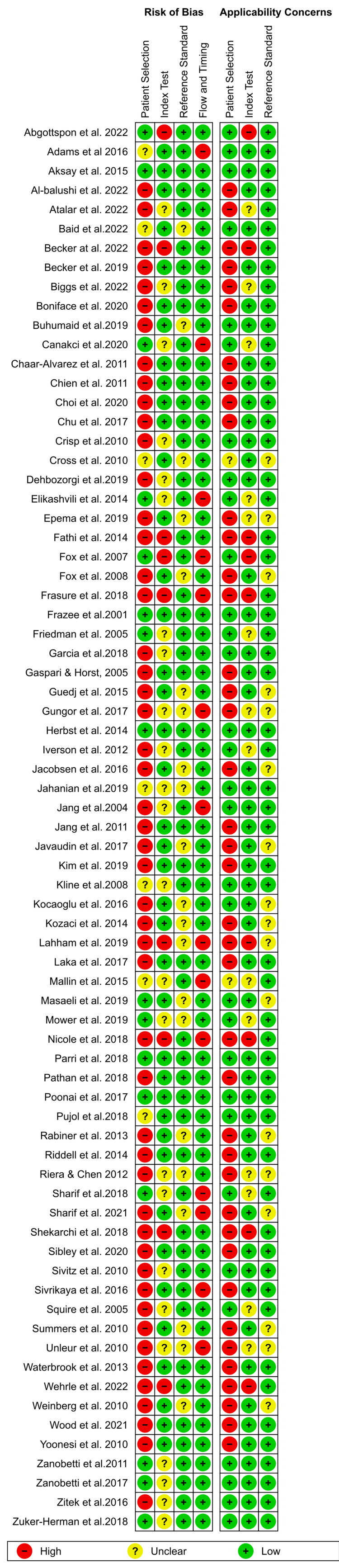
Risk of bias assessment according to QUADAS-2 tool Elikashvili et al. [16], Gungor et al. [17], Nicole et al. [18], Becker et al. [19], Fox et al. [20], Atalar et al. [21], Fathi et al. [22], Mallin et al. [23], Sharif et al. [24], Fox et al. [25], Abgottspon et al. [26], Gaspari & Horst [27], Pathan et al. [28], Guedj et al. [29], Herbst et al. [30], Javaudin et al. [31], Sibley et al. [32], Al-Balushi et al. [33], Riddell et al. [34], Boniface et al. [35], Unleur et al. [36], Becker et al. [37], Biggs et al. [38], Jang et al. [39], Frasure et al. [40], Shekarchi et al. [41], Sharif et al. [42], Summers et al. [43], Wehrle et al. [44], Yoonesi et al. [45], Kim et al. [46], Chu et al. [47], Lahham et al. [48], Jacobsen et al. [49], Mower et al. [50], Squire et al. [51], Adams et al. [52], Iverson et al. [53], Sivitz et al. [54], Friedman et al. [55], Cross et al. [56], Chien et al. [57], Waterbrook et al. [58], Sivrikaya et al. [59], Poonai et al. [60], Laka et al. [61],Chaar-Alvarez et al. [62], Epema et al. [63], Kozaci et al. [64], Wood et al. [65], Kocaoglu et al. [66], Aksay et al. [67], Kozaci et al. [68], Masaeli et al. [69], Choi et al. [70], Parri et al. [71], Rabiner et al. [72], Riera & Chen [73], Weinberg et al. [74], Canakci et al. [75], Garcia et al. [76], Jang et al. [77], Crisp et al. [78], Jahanian et al. [79], Frazee et al. [80], Dehbozorgi et al. [81], Kline et al. [82], Pujol et al. [83], Zitek et al. [84], Zuker-Herman et al. [85], Baid et al. [86], Zanobetti et al. [87], Zanobetti et al. [88], and Buhumaid et al. [89].

**Figure 3:**
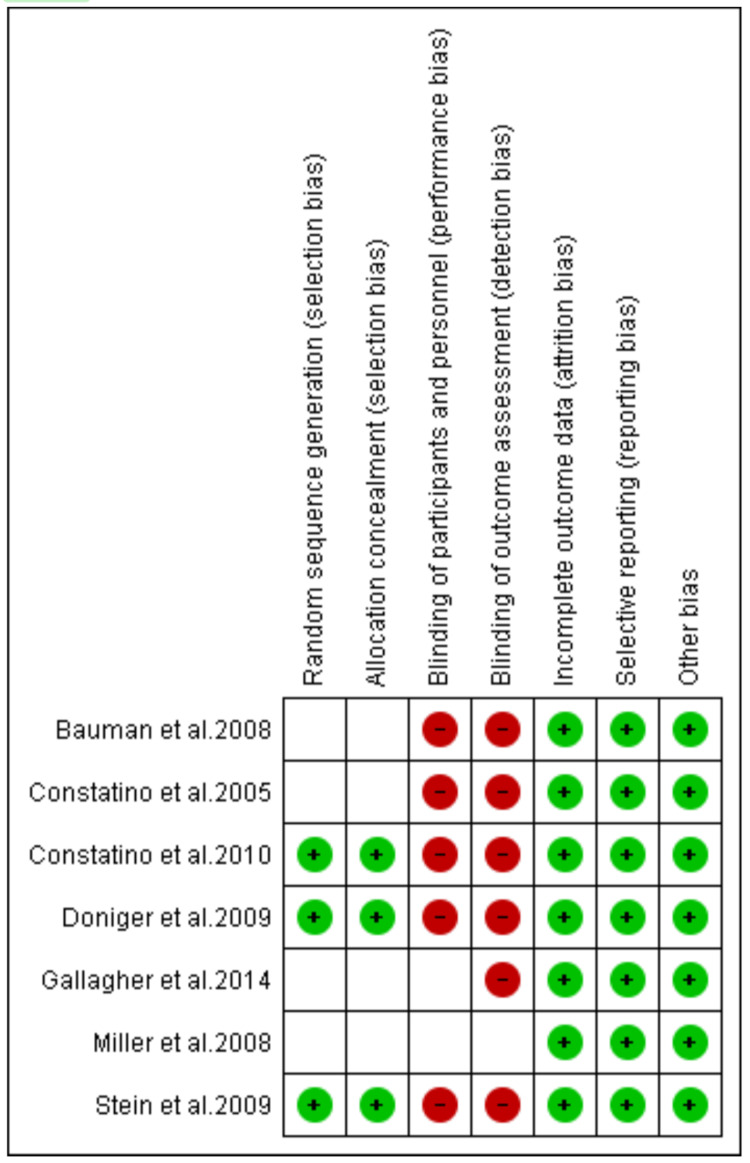
Risk of bias summary. Costantino et al. [90], Costantino et al. [91], Doniger et al. [92], Stein et al. [93], Bauman et al. [94], Miller et al. [95], and Gallagher et al. [96].

The full risk of bias assessment according to the QUADAS-2 tool is summarized in Figure 2, while the assessment according to the RoB is outlined in Figure 3. For the QUADAS scores, a high risk of bias under the patient selection was outlined when a convenience sample was used. Furthermore, studies that had more than one reference standard were regarded to have a high risk of flow and timing bias.

POCUS as a Diagnostic Tool in the ED

In this study, the diagnostic use of POCUS in the ED was categorized according to the body region. The pooled analysis showed that abdominal POCUS had a sensitivity and specificity of 65.61% and 88.86% for appendicitis, 81.74% and 74.09% for hydronephrosis, 93.15% and 81.80% for small bowel obstruction (SBO), and 74.73% and 95.56% for cholecystitis, respectively. The pooled analysis also showed that ocular POCUS was 93.66% sensitive and 90.90% specific when diagnosing retinal detachment in the ED.

In addition, our analysis suggested that soft-tissue POCUS aids in the diagnosis of abscess with a sensitivity of 94.75% and a specificity of 85.15%, and in identifying foreign bodies with a sensitivity of 66.7% and a specificity of 96.6%. Furthermore, musculoskeletal POCUS demonstrated a sensitivity and specificity of 93.11% and 94.94% for clavicle fractures, 96.76% and 94.94% for distal forearm fractures, 94.20% and 92.36% for metacarpal fractures, and 90.78% and 97.06% for skull fractures.

On the other hand, subgroup analyses on the diagnostic value of vascular POCUS showed that two-point compression POCUS diagnoses deep vein thrombosis (DVT) with a sensitivity of 88.85% and a specificity of 96.29%, while three-point compression POCUS is 86.93% sensitive and 92.41% specific. Moreover, lung POCUS aids the diagnosis of pleural effusion with a sensitivity and specificity of 90.56% and 96.81%, respectively. These pooled analyses are outlined in Table 3.

**Table 3:** Pooled diagnostic accuracy of POCUS. POCUS = point-of-care ultrasound; CI = confidence interval

Region of POCUS assessment	Condition	Number of studies	Pooled Sensitivity (95% CI)	Pooled Specificity (95% CI)
Abdominal	Appendicitis	11	65.31 (52.68–77.93)	88.86 (83.16–94.56)
	Hydronephrosis	8	81.74 (76.57–86.91)	74.09 (59.90–88.29)
	Small bowel obstruction	6	93.15 (88.76–97.54)	81.80 (69.29–94.31)
	Cholecystitis	4	74.73 (58.06–91.41)	95.56 (92.15–98.97)
Ocular	Retinal detachment	5	93.66 (87.30–100)	90.90 (86.71–95.10)
Soft tissue	Abscess	5	94.75 (90.64–98.87)	85.15 (72.21–98.09)
	Foreign bodies	1	66.7 (34.8–90.1)	96.6 (91.6–99.1)
Musculoskeletal	Clavicle fractures	4	93.11 (87.05–99.16)	94.94 (89.48–100)
	Distal forearm fractures	7	96.76 (94.59–98.94)	94.94 (92.88–97.02)
	Metacarpal fractures	4	94.20 (89.29–99.11)	92.36 (85.92–98.80)
	Skull fractures	6	90.78 (86.42–95.14)	97.06 (94.42–99.69)
Vascular	Deep vein thrombosis	6 (two-point POCUS)	88.85 (79.43–98.27)	96.29 (93.39–99.19)
		6 (three-point POCUS)	86.93 (76.92–96.93)	92.41 (88.03–96.78)
Lung	Pleural effusion	4	90.56 (75.47–100)	96.81 (94.94–98.69)

POCUS for the Guidance of Procedures in the ED

In this study, we analyzed the use of POCUS in guiding central venous insertion and peripheral venous cannulation. The pooled outcomes showed that the success rate for peripheral venous access was considerably higher when guided by POCUS compared to the traditional method (p = 0.02). However, the interstudy heterogeneity was high (80%). Similarly, the pooled analysis suggested that the number of skin punctures required for peripheral venous access was significantly less when using POCUS for guidance compared to the traditional techniques (p = 0.01). However, the heterogeneity was substantial (98%). On the other hand, our meta-analyses showed that ultrasound-guided and traditional methods of peripheral venous access are equally effective and safe, as demonstrated by the time taken to achieve successful cannulation and complication rates.

Regarding the use of POCUS to guide central venous catheter (CVC) insertion, we found no significant difference in the rate of procedure-related complications between the ultrasound-guided and traditional methods. However, one of the studies, including pediatric patients only, reported that the rate of successful CVC insertion was higher when using POCUS guidance (OR = 13.1; 95% CI = 2.9-59). Similarly, Miller and colleagues reported that among patients undergoing CVC insertion, POCUS guidance significantly reduced the time to successful insertion and the number of skin punctures required compared to the landmark technique (p < 0.0001). The outcomes of POCUS as a tool guiding procedures in the ED are summarized in Table 4.

**Table 4:** Meta-analytic outcomes of ultrasound-guided procedures compared to traditional methods. CI = confidence interval; OR = odds ratio; MD = mean differences

Procedure	Outcome	Number of studies	Effect size (95% CI)	P-value	Heterogeneity (I^2^)
Peripheral venous access	Overall success rate	4	OR = 5.88 (1.32–26.28)	0.02	80%
	Number of punctures	5	MD = -1.17 (-2.06–-0.27)	0.01	98%
	Time to cannulation	5	MD = -4.93 (-12.96–3.09)	0.23	93%
	Complications	5	OR = 0.41 (0.13–1.33)	0.14	14%
Central venous access	Complications	2	OR = 0.82 (95% 0.41–1.64)	0.57	0%

Discussion

This systematic review and meta-analysis has summarized the procedural and diagnostic use of POCUS in the ED. The pooled analysis showed that in most conditions, POCUS has a high sensitivity, meaning it can help emergency physicians (EPs) rule out a condition, and for other conditions, it has high specificity, meaning it can rule in a diagnosis [97]. Furthermore, our statistical analyses suggest that POCUS can improve the success rate of peripheral and central venous access and reduce the number of skin punctures compared to conventional methods.

Abdominal ultrasound is an imaging test that helps healthcare providers diagnose or rule out health conditions in the abdominal areas. In this review, we analyzed the use of abdominal POCUS in diagnosing appendicitis, hydronephrosis, SBO, and cholecystitis. Our pooled analyses showed that POCUS has a moderate sensitivity and a high specificity in diagnosing appendicitis. These findings align with what was already recorded in previous studies. Nicole and colleagues found that among pediatric patients only, POCUS had a limited sensitivity (53%) but a relatively high specificity (82%) for appendicitis [18]. Similarly, Lee and Yun found that EP-performed POCUS had a higher specificity for appendicitis diagnosis than sensitivity (91% vs. 84%) [98]. Considering these findings, it seems that there is inconclusive evidence on the use of POCUS to rule out appendicitis in the ED; however, POCUS has the potential to be used in ruling in this condition.

The usefulness of POCUS in diagnosing hydronephrosis was also evaluated in the present study. The pooled analyses showed that POCUS has a high sensitivity and moderate specificity for diagnosing hydronephrosis. However, the diagnostic accuracy varied from study to study. In their research, Pathan and colleagues reported that when using a CT scan as the reference standard, POCUS was highly specific (94.6%) in diagnosing moderate-to-severe hydronephrosis but had an inferior sensitivity (34.2%) [28]. This finding is also witnessed in other studies where CT scans were used as the reference standard [33]. On the other hand, Javaudin and colleagues found that EP without any previous POCUS skills can rule out hydronephrosis in the ED with satisfactory sensitivity (100%) [31]. This contradictory information suggests that there is still a gap in the use of POCUS to diagnose hydronephrosis in the ED, and more randomized trials are required. However, the contradiction observed can be related to the fact that Jauvadin and colleagues used radiologist-performed ultrasound as the reference standard rather than a CT scan which is considered the gold standard imaging modality for hydronephrosis [34].

SBO symptoms are frequently observed in ED patients. Typically, the gold standard diagnosis for this condition before hospital admission is a CT scan. However, currently, the use of ultrasound at the patient’s bedside has emerged as an intriguing concept. In our study, we found that POCUS had a high sensitivity and specificity for SBO, suggesting that POCUS can play a vital role in ruling out and diagnosing SBO in the ED. However, some studies have reported contradictory information. Becker and colleagues reported that although POCUS was highly sensitive (88%) for SBO, it was less specific (54%) [37]. The difference cited in this study can be attributed to the fact that most of their sonographers were highly inexperienced when it came to using POCUS. Additionally, our meta-analysis showed that POCUS has moderate sensitivity but is highly specific in diagnosing cholecystitis in the ED. Therefore, our findings suggest that POCUS might be a reliable tool for ruling in cholecystitis in the ED.

The Diagnostic Utility of Ocular POCUS

In the ED, patients can present with various ocular emergencies, ranging from simple conjunctivitis to sight-threatening diseases. Due to limited access to ophthalmology in some settings, these emergencies may burden the EP to make swift decisions [99]. One of the common eye complications presenting to the ED is retinal detachment; therefore, it is worth noting whether POCUS may aid the EP in diagnosing this condition. Our statistical analyses found that POCUS is highly sensitive and specific when diagnosing retinal detachment in the ED. This is further reinforced by a 2019 meta-analysis that found that ocular POCUS performed by EP had a sensitivity and specificity of 94% and 91% for retinal detachment [100]. Therefore, it is evident that POCUS can accurately diagnose retinal detachment in the ED. Furthermore, one of the studies reported a high sensitivity and specificity for vitreous hemorrhage (81.9% and 82.3%, respectively) and a very high specificity for vitreous detachment (96.6%) [48].

The Diagnostic Utility of Soft Tissue POCUS

Skin and soft tissue infections (SSTIs) are among the most common ED complications. In our research, we found that among patients with signs of SSTI, POCUS was 95% sensitive and 85% specific in diagnosing abscesses. This finding aligns with a previous systematic review, which recorded a pooled sensitivity and specificity of 96% and 83%, respectively, for diagnosis of abscess [101]. Furthermore, a 2016 meta-analysis demonstrated similar test characteristics [102]. These findings suggest that among patients presenting to the ED with SSTIs, POCUS is sufficient to diagnose abscesses. In addition, data from one study indicated that POCUS can be used to rule in foreign bodies in the ED due to its high specificity [55].

The Diagnostic Utility of Musculoskeletal POCUS

Complaints about musculoskeletal pain are very common in the ED. In our study, we investigated the diagnostic accuracy of POCUS in diagnosing clavicle, distal forearm, metacarpal, and skull fractures. The analyses have shown that POCUS is highly sensitive and specific when diagnosing these musculoskeletal fractures in the ED. These findings concur with the previous studies on the use of POCUS to diagnose musculoskeletal fractures [103]. Therefore, it is evident that POCUS can accurately diagnose clavicle, distal forearm, metacarpal, and skull fractures among adult and pediatric patients in the ED. Furthermore, evidence suggests that POCUS can aid in diagnosing tendon injuries such as Achilles, patellar tendons, and quadriceps in the ED [104-106]. However, the high diagnostic accuracy of POCUS for musculoskeletal fractures does not mean that it can replace the need for subsequent X-rays but can be used as a complementary imaging tool or as an alternative in cases where X-rays are not immediately available.

The Diagnostic Utility of Vascular POCUS

DVT is a life-threatening vascular condition affecting patients of all ages. Therefore, rapid and accurate diagnosis of this condition is crucial because research has shown that one-third of DVTs that are left untreated progress to significant pulmonary embolism [107]. The gold standard test for DVT is contrast venography; however, vascular POCUS is increasingly being employed in the ED to assess this condition [8,108]. Currently, there are two kinds of POCUS techniques used to evaluate DVT in the lower extremities. The first technique is the two-point compression which involves testing the compressibility of the common femoral vein (CFV) and the popliteal vein (PV), and the other technique is the three-point compression which involves the compression of CFV, PV, and the superficial femoral vein (SFV). In this meta-analysis, we found that two-point and three-point POCUS techniques had similar test characteristics when evaluating DVT in the ED. These findings are corroborated by a previous meta-analysis of 2,372 patients which found that the two-point POCUS had a similar pooled sensitivity and specificity as the three-point POCUS [109]. Given that the test characteristics were high, POCUS can be used to accurately diagnose lower extremity DVT in the ED.

The Diagnostic Utility of Lung POCUS

Shortness of breath (medically known as dyspnea) and chest pain are common complaints in the ED. The initial management of these complaints can be challenging because of the differential diagnoses, which are often life-threatening conditions in need of urgent identification and management [110]. Generally, a chest radiograph (CXR) is the initial diagnostic tool for patients with dyspnea and chest pain. However, previous research has shown that CXR has limited sensitivity and specificity, raising questions about its diagnostic utility [87,111]. As a result, the use of POCUS in different clinical settings to assess the causes of dyspnea and chest pain is rapidly growing. In our study, we found that among dyspneic patients presenting to the ED, POCUS had a sensitivity and specificity of 91% and 97%, respectively, for the diagnosis of pleural effusion. Therefore, POCUS seems to be a feasible diagnostic tool that can narrow down the diagnosis of pleural effusion. Given the benefits of POCUS in reducing healthcare costs, enhancing fast care delivery, and radiation-free testing, our findings suggest that POCUS can be incorporated in the ED to diagnose pleural effusion in patients presenting with dyspnea and chest pain.

POCUS for Procedure Guidance in the ED

In the ED, POCUS can be used to guide several procedures; however, in this review, we investigated the use of POCUS in guiding peripheral venous and central venous access. Patients with difficult peripheral venous access usually require multiple attempts and perhaps central venous access, resulting in increased time and resource use in the ED. Therefore, it is worth studying whether ultrasound guidance may help improve peripheral venous access. In our study, we found that ultrasound guidance significantly improved the success rate and reduced the number of skin punctures during peripheral venous access compared to the traditional methods. However, the heterogeneity between studies was substantial, suggesting outcome variation. Furthermore, we found that ultrasound guidance does not reduce the time taken for successful cannulation compared to the traditional techniques. Therefore, further high-quality randomized trials are required to support the potential benefit of POCUS in guiding peripheral venous access. Similarly, evidence suggests that POCUS may be beneficial in performing CVC insertion more rapidly and in fewer attempts; however, this finding requires further investigation.

Limitations

The current study has several limitations that should be accounted for when interpreting the findings. First, we observed substantial heterogeneity in several meta-analyses. However, we used the random-effect model to counter this heterogeneity and provide conservative results. Second, we only included studies published in English from the year 2000 onward; hence, it is possible that selection bias was introduced in our research. Finally, most studies in this review were non-randomized, which might have introduced the confounding bias witnessed in such studies.

## Conclusions

This systematic review and meta-analysis shows that POCUS has increasingly been utilized in the ED over the past two decades. It also indicates that POCUS in the ED can accurately diagnose clinical conditions such as SBO, retinal detachment, abscess, clavicle fractures, distal forearm fractures, metacarpal fractures, skull fractures, pleural effusion, and DVT. In addition, we have demonstrated that POCUS can aid in ruling in appendicitis, foreign bodies, and cholecystitis due to its high specificity. Furthermore, POCUS can potentially increase the success rate of peripheral vein and central venous access and decrease the number of skin punctures; however, these findings require further investigations in high-quality randomized trials.

## Figures and Tables

**Figure 4:**
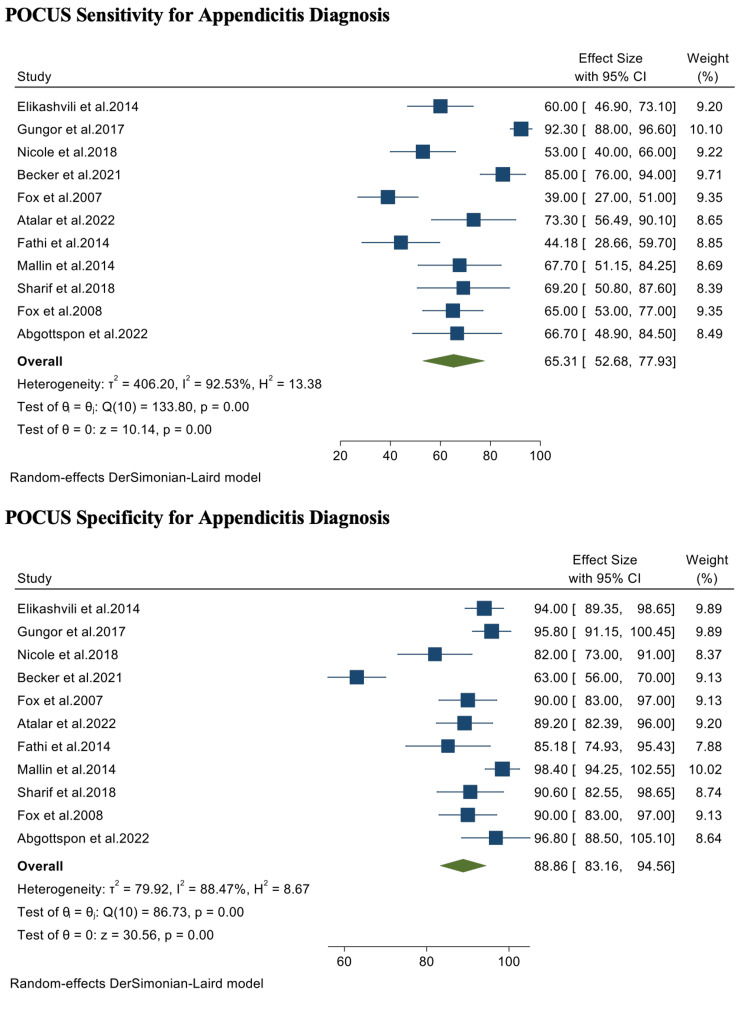
Point-of-care ultrasound sensitivity and specificity for appendicitis diagnosis. Elikashvili et al. [16], Gungor et al. [17], Nicole et al. [18], Becker et al. [19], Fox et al. [20], Atalar et al. [21], Fathi et al. [22], Mallin et al. [23], Sharif et al. [24], Fox et al. [25], and Abgottspon et al. [26].

**Figure 5:**
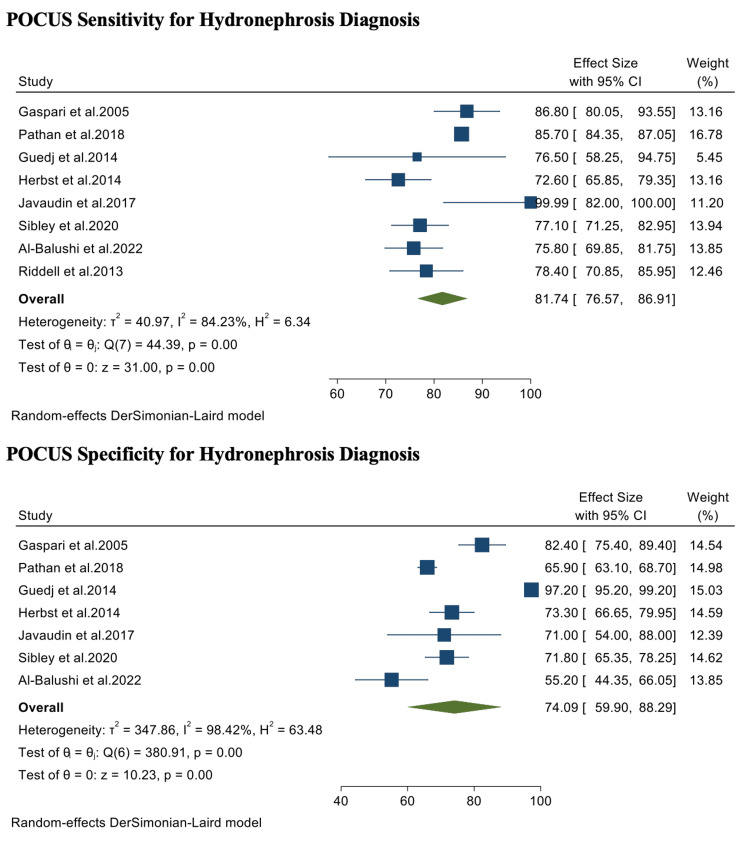
Point-of-care ultrasound sensitivity and specificity for hydronephrosis diagnosis. Gaspari & Horst [27], Pathan et al. [28], Guedj et al. [29], Herbst et al. [30], Javaudin et al. [31], Sibley et al. [32], Al-Balushi et al. [33], and Riddell et al. [34].

**Figure 6:**
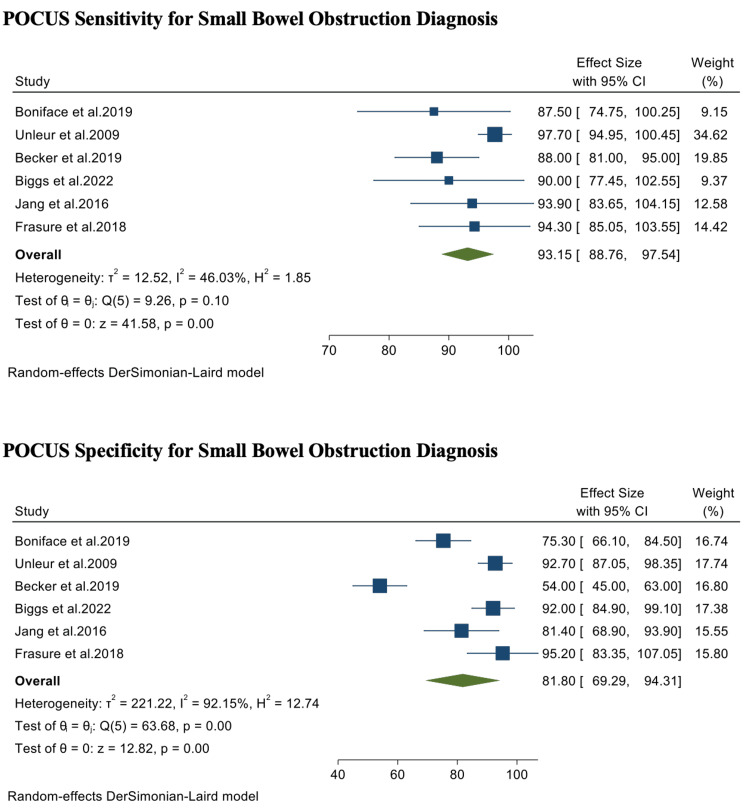
Point-of-care ultrasound sensitivity and specificity for small bowel obstruction diagnosis. Boniface et al. [35], Unleur et al. [36], Becker et al. [37], Biggs et al. [38], Jang et al. [39], and Frasure et al. [40].

**Figure 7:**
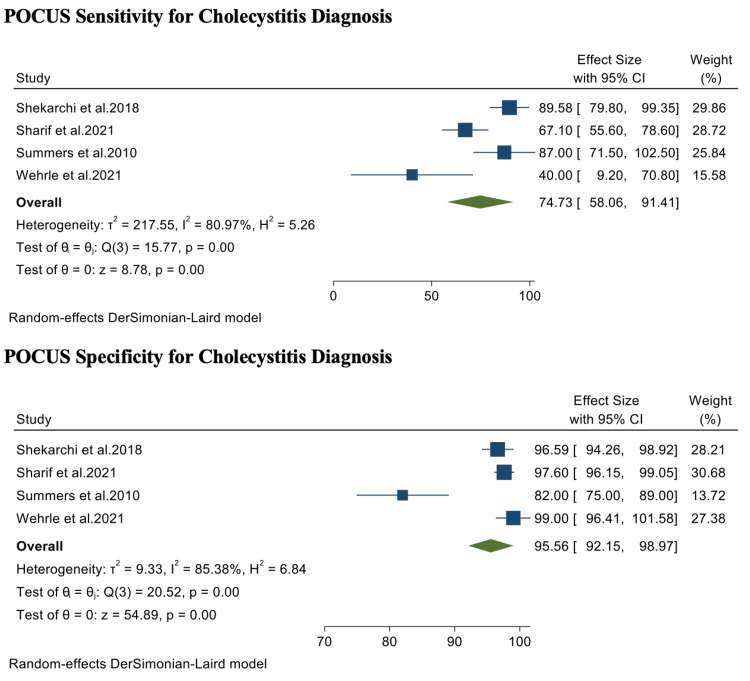
Point-of-care ultrasound sensitivity and specificity for cholecystitis diagnosis. Shekarchi et al. [41], Sharif et al. [42], Summers et al. [43], and Wehrle et al. [44].

**Figure 8:**
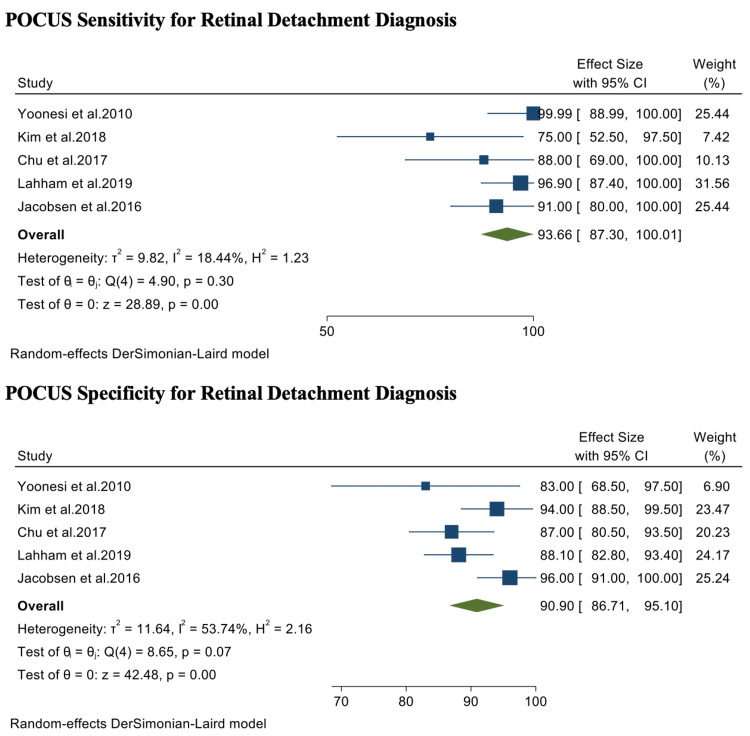
Point-of-care ultrasound sensitivity and specificity for retinal detachment diagnosis. Yoonesi et al. [45], Kim et al. [46], Chu et al. [47], Lahham et al. [48], and Jacobsen et al. [49].

**Figure 9:**
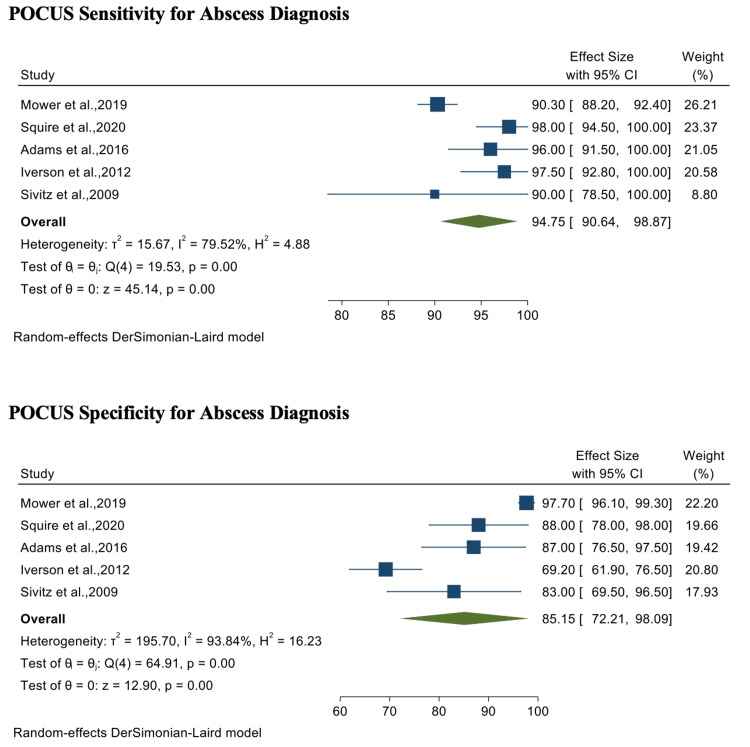
Point-of-care ultrasound sensitivity and specificity for abscess diagnosis. Mower et al. [50], Squire et al. [51], Adams et al. [52], Iverson et al. [53], and Sivitz et al. [54].

**Figure 10:**
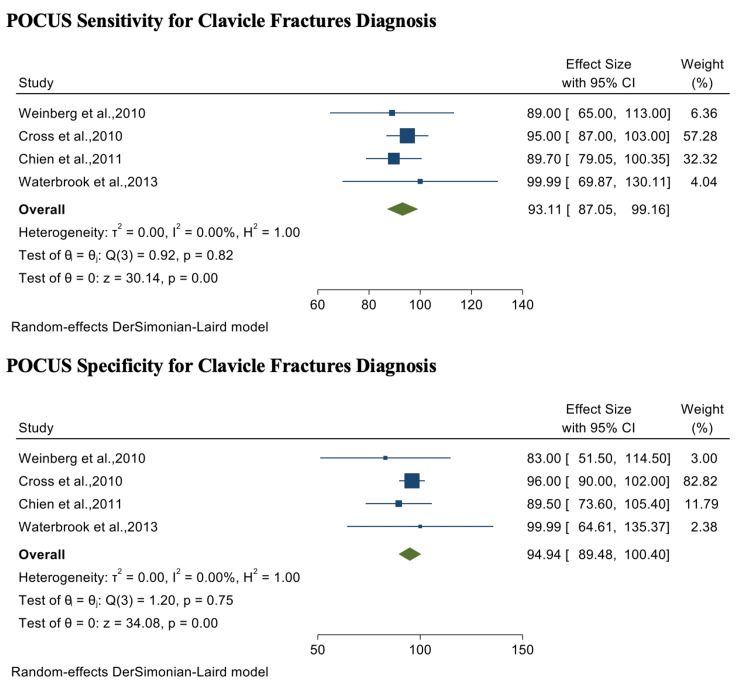
Point-of-care ultrasound sensitivity and specificity for clavicle fracture diagnosis. Cross et al. [56], Chien et al. [57], Waterbrook et al. [58], and Weinberg et al. [74].

**Figure 11:**
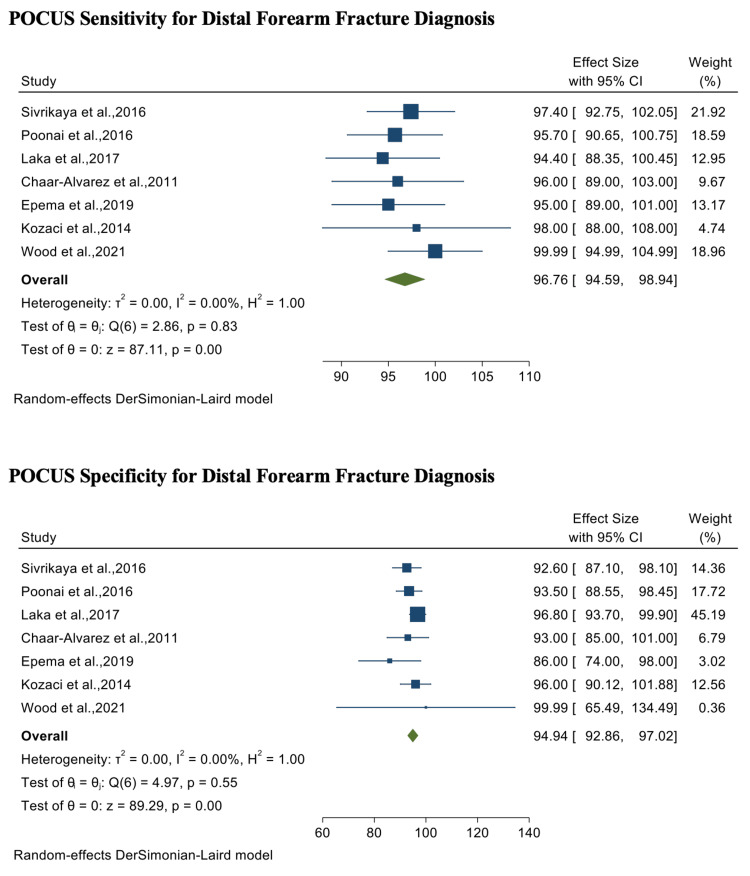
Point-of-care ultrasound sensitivity and specificity for distal forearm fracture diagnosis. Sivrikaya et al. [59], Poonai et al. [60], Laka et al. [61], Chaar-Alvarez et al. [62], Epema et al. [63], Kozaci et al. [64], and Wood et al. [65].

**Figure 12:**
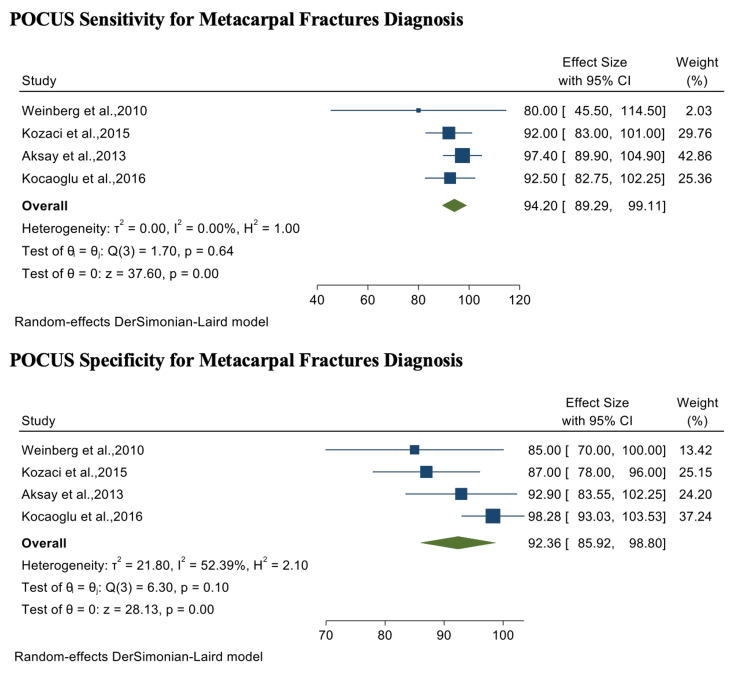
Point-of-care ultrasound sensitivity and specificity for metacarpal fracture diagnosis. Kocaoglu et al. [66], Aksay et al. [67], Kozaci et al. [68], and Weinberg et al. [74].

**Figure 13:**
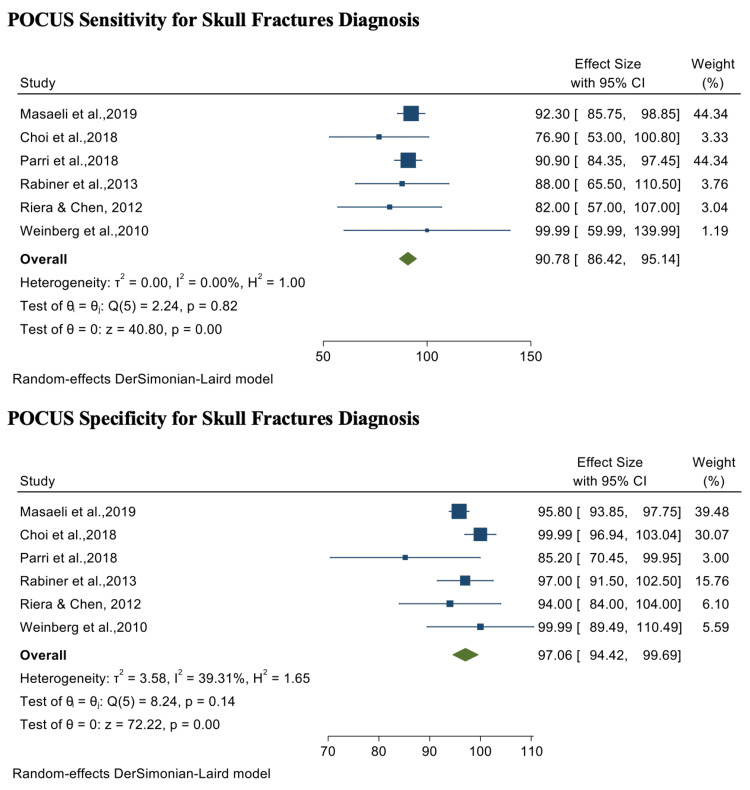
Point-of-care ultrasound sensitivity and specificity for skull fracture diagnosis. Masaeli et al. [69], Choi et al. [70], Parri et al. [71], Rabiner et al. [72], Riera & Chen [73], and Weinberg et al. [74].

**Figure 14:**
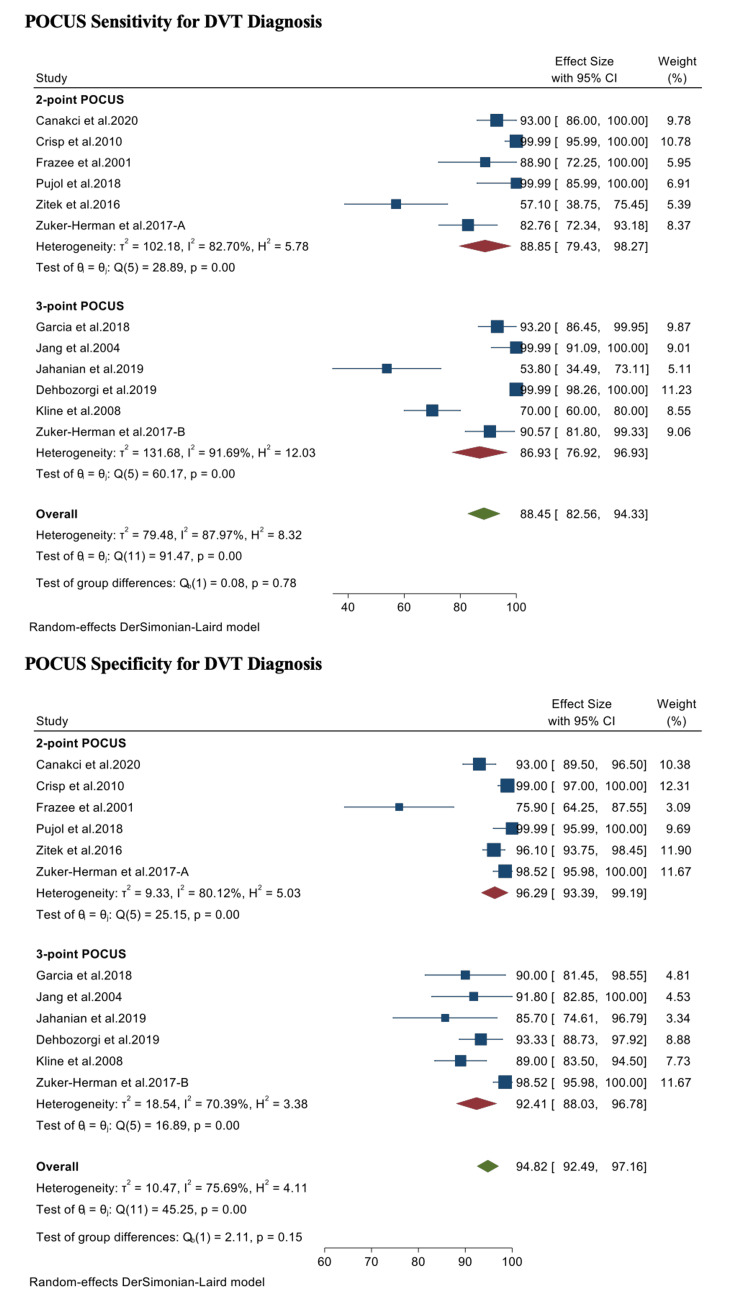
Point-of-care ultrasound sensitivity and specificity for deep vein thrombosis diagnosis. Canakci et al. [75], Garcia et al. [76], Jang et al. [77], Crisp et al. [78], Jahanian et al. [79], Frazee et al. [80], Dehbozorgi et al. [81], Kline et al. [82], Pujol et al. [83], Zitek et al. [84], and Zuker-Herman et al. [85].

**Figure 15:**
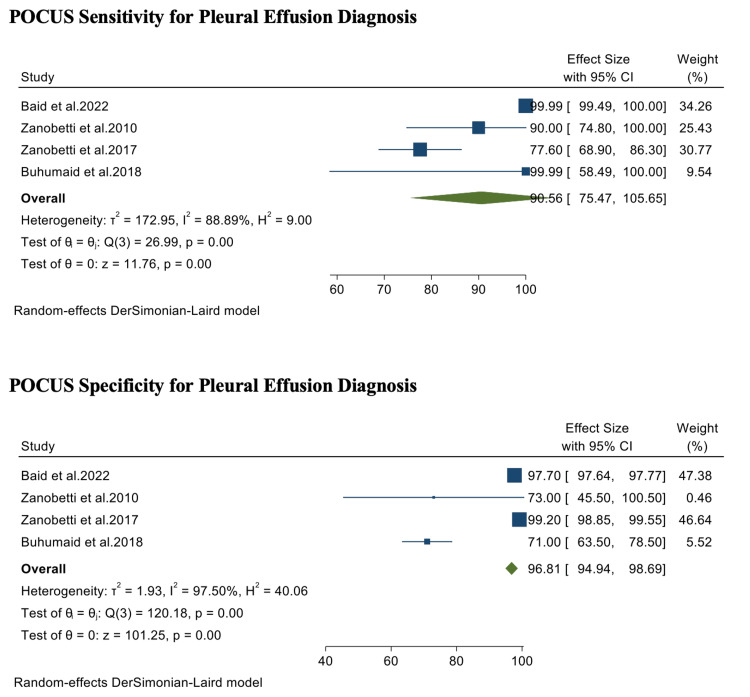
Point-of-care ultrasound sensitivity and specificity for pleural effusion diagnosis. Baid et al. [86], Zanobetti et al. [87], Zanobetti et al. [88], and Buhumaid et al. [89].

**Figure 16:**
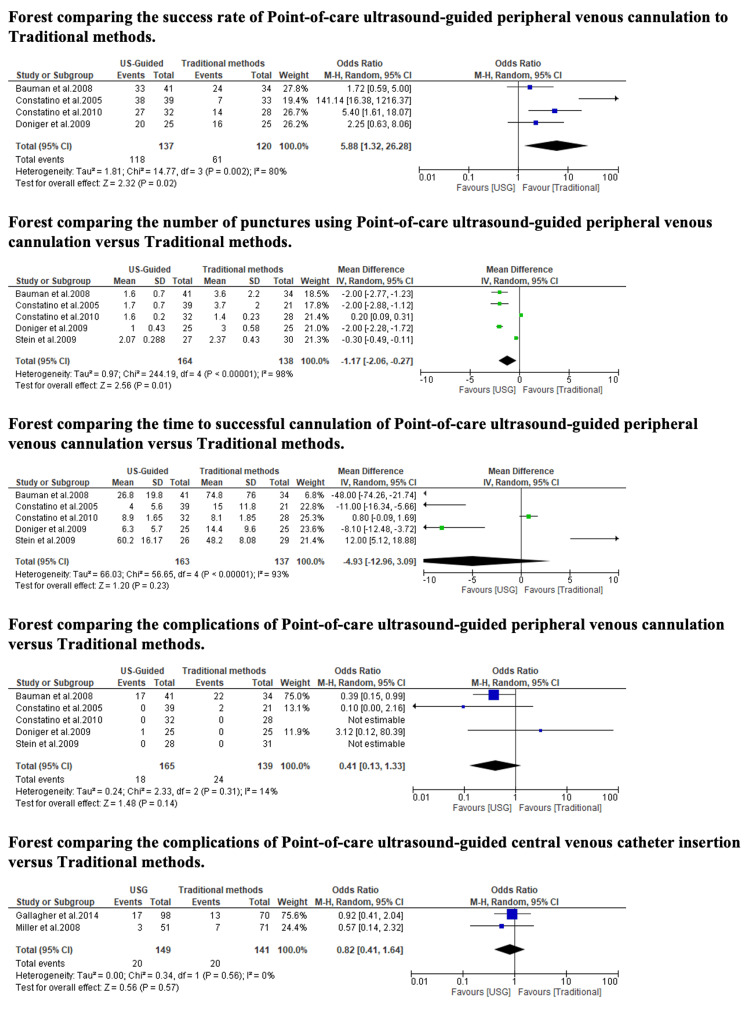
Forest plots comparing the efficacy and safety of point-of-care ultrasound-guided versus traditional vascular cannulation techniques. Costantino et al. [90], Costantino et al. [91], Doniger et al. [92], Stein et al. [93], Bauman et al. [94], Miller et al. [95], and Gallagher et al. [96].
